# Transmission Line Vibration Damper Detection Using Multi-Granularity Conditional Generative Adversarial Nets Based on UAV Inspection Images

**DOI:** 10.3390/s22051886

**Published:** 2022-02-28

**Authors:** Wenxiang Chen, Yingna Li, Zhengang Zhao

**Affiliations:** 1 Faculty of Information Engineering and Automation, Kunming University of Science and Technology, Kunming 650500, China; 20182204169@stu.kust.edu.cn (W.C.); 20192204211@stu.kust.edu.cn (Z.Z.); 2 Computer Technology Application Key Lab of the Yunnan Province, Kunming 650500, China

**Keywords:** power transmission lines, vibration dampers detection, unmanned aerial vehicle (UAV), conditional generative adversarial nets (CGAN), Monte Carlo search (MCS)

## Abstract

The vibration dampers can eliminate the galloping phenomenon of transmission lines caused by the wind. The detection of vibration dampers based on visual technology is an important issue. Current CNN-based methods struggle to meet the requirements of real-time detection. Therefore, the current vibration damper detection work has mainly been carried out manually. In view of the above situation, we propose a vibration damper detection-image generation model called DamperGAN based on multi-granularity Conditional Generative Adversarial Nets. DamperGAN first generates a low-resolution detection result image based on a coarse-grained module, then uses Monte Carlo search to mine the latent information in the low-resolution image, and finally injects this information into a fine-grained module through an attention mechanism to output high-resolution images and penalize poor intermediate information. At the same time, we propose a multi-level discriminator based on the multi-task learning mechanism to improve the discriminator’s discriminative ability and promote the generator to output better images. Finally, experiments on the self-built DamperGenSet dataset show that the images generated by our model are superior to the current mainstream baselines in both resolution and quality.

## 1. Introduction

The galloping phenomenon is prone to occur when high-voltage transmission lines are subjected to wind forces. Long-term vibration leads to metal fatigue damage at the suspension, which leads to serious accidents such as wire breakage and tower collapse. The use of vibration dampers on high-voltage transmission lines can reduce the vibration of the wires caused by the wind, thereby reducing the probability of accidents. The aim of a vibration damper detection task is to provide the position of the vibration damper in the image. It is the basis for other tasks such as vibration damper corrosion inspection and mobile inspection. At present, this problem has attracted the attention of researchers studying smart grids and other fields [1,2].

In recent years, UAV-related technology has become one of the fastest developing directions in the field of science and technology. UAVs have the advantages of simple operation, easy portability, and low use cost that other aircraft such as helicopters do not have [3]. The UAV array network composed of sensor networks [4] can quickly complete tasks such as object recognition [5] and crop yield estimation [6]. While drones bring convenience to human activities, they also bring some negative effects [7]. Examples include the communication security issues of UAVs [8] and the noise pollution generated during flight, which threatens the safety of birds [9]. Current transmission line inspection tasks are still mainly performed manually. Therefore, the use of UAVs for power line inspection is an issue worth researching, and there have been a number of relevant studies [10]. This paper focuses on the detection of line vibration dampers using aerial images obtained by UAVs.

In early work, the use of image processing algorithms to improve the visual perception of vibration dampers in images was the most common method. Usually, researchers use an appropriate feature extraction operator to detect vibration dampers [11]. In addition, there are studies that combine machine learning algorithms to improve the level of automation [1], and such methods were also the main direction of early research. However, traditional methods generally have the problem of a low detection accuracy.

In recent years, with the renewed popularity of deep learning technology, multiple algorithms represented by convolutional neural networks (CNNs) have emerged, providing new energy to the task of vibration damper detection. Such methods can obtain an end-to-end model through iterative training on the dataset, and only one input is required to obtain an output with excellent detection accuracy in subsequent use. Although CNN-like algorithms have high detection accuracy, they still have the problem of a long training time, and the effect is limited by the size of the dataset. We hope to obtain a method with low resource requirements and a high detection effect.

Benefiting from the rapid development of deep learning, image generation algorithms represented by GAN [12] have become one of the research hotspots in the field of computer vision. CGAN [13] through additional auxiliary conditions and the use of common data annotations in the field of object detection can generate vibration damper detection images.

**Figure 1:**
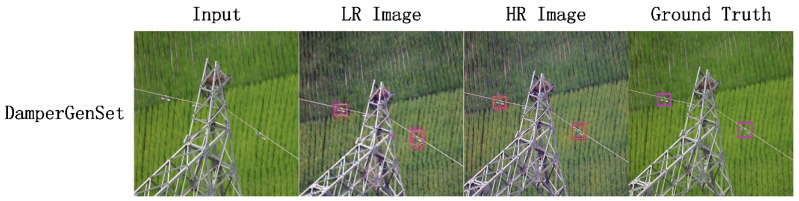
Test examples of our model on DamperGenSet.

In view of the research status of the field of vibration damper detection, as shown in Figure 1, we proposed a model for line vibration damper detection image generation that is based on a conditional generative adversarial network. The main contributions of this paper are as follows:

- On the basis of an improved conditional generative adversarial network, we proposed a framework for vibration damper detection image generation named DamperGAN. The framework contains a two-stage generator and a multi-scale discriminator.
- In order to generate high-resolution vibration damper detection images, we used a coarse-to-fine generation method. At the same time, an attention mechanism and a penalty mechanism based on Monte Carlo search were introduced into the generator to provide enough semantic information for image generation to improve the edge information of the image.
- In order to improve the high-resolution images generated by the discriminator, we proposed a multi-level discriminator structure based on the parameter sharing mechanism, so that the entire discriminator pays attention to both semantic information and underlying feature information.
- Aiming to address the problem of no public dataset in the field of vibration damper detection, we established a dataset named DamperGenSet that is based on real optical images from UAV aerial photography. Through comparison with the experimental results of multiple state-of-the-art models on the DamperGenSet dataset, we prove that our proposed DamperGAN has certain advantages.

The remainder of this article is organized as follows. Section 2 briefly introduces related work on vibration damper detection and image generation. Section 3 introduces the basic knowledge used in this article. In Section 4, we introduce the details of DamperGAN. In Section 5, we introduce the dataset, experimental details, and a series of comparative experiments. Section 6 provides a brief summary of the work in this article.

## 2. Related Work

In this section, we focus on related work on vibration damper detection using image technology, including research using traditional techniques and deep learning techniques.

### 2.1. Damper Detection

In recent years, there has been a certain amount of research on vibration dampers for transmission lines. In the early days, the research on vibration dampers was mainly based on image processing algorithms and edge detection models. In recent years, the convolutional neural network represented by AlexNet [14] has developed rapidly, providing new solutions for the field of object detection, and there have been increasingly more studies on CNN-based vibration damper detection.

The simplest method in the traditional algorithm is to use the statistical properties of basic colors in an image, such as the use of histogram technology for vibration damper corrosion detection [15]. This is also a detection method that improves the visibility of the vibration damper in an image via color space transformation [16]. Huang et al. [17] used grayscale processing, edge detection, threshold segmentation, morphological processing, and other technologies to calculate the rusted area ratio of the vibration damper to determine the degree of corrosion of the vibration damper and carried out displacement detection. Pan et al. [11] used the edge extraction operator to estimate the damage degree of the vibration damper. Extracting the edge of the vibration damper is also an effective detection method. For example, the Canny operator and Hough transform were used to detect the vibration damper, and the displacement distance of the vibration damper was calculated on the basis of the edge information [18]. Miao et al. [19] used the wavelet transform on the vibration damper detection problem. In a study by Wu et al. [2], on the basis of the helicopter aerial image, the authors used the Snake model to extract the edge of the vibration damper. Edge detection operators combined with machine learning algorithms were the most robust method before CNNs. Jin et al. [20,21] performed vibration damper detection and classification tasks using Harr features and cascaded Adaboost classifiers based on UAV aerial images.

At present, CNN-based research has become the main work conducted in the field of vibration damper detection of transmission lines, for example, YOLOv4 [1,22], based on the one-stage class method; Faster R-CNN [16,23], based on the two-stage class method; and research based on Cascade R-CNN [24]. However, the amount of work on vibration dampers is currently relatively small. Additionally, there are CNN-based studies on other power line components and foreign objects. For example, Faster R-CNN is used to detect the shape change of insulator strings [25], insulators [26], and icing on power lines [27]. Mask R-CNN is used to detect foreign objects such as bird nests and balloons on transmission lines [28], insulator defect detection of high-speed rail power transmission lines [29], and infrared imaging equipment used to detect the humidity of insulators [30]. Usually, these studies are merely simple applications of power component datasets; most of the studies lack targeted modification for specific environments and scenarios, and the solutions provided are mostly skill stacking. However, these studies usually only use CNNs to perform model iteration on the dataset, without targeting the particularity of the environment in which the object is located. In addition, the performance of the model is very dependent on the size of the training set.

### 2.2. Image Generation

With the rise of deep learning, the field of image generation has also been given new research directions. Variational autoencoder (VAE) [31] is the earliest model, which is based on probability maps in the field of image generation. Some researchers [32] added an attention mechanism to VAE and proposed a high-quality image generation model based on DRAW. In [33], researchers proposed a mechanism for generating images using visual features named Attribute2Image, which is able to synthesize images with a separate foreground and background.

In recent years, the advent of adversarial generative networks (GAN) [12] has provided new vitality to image generation. GAN uses the mechanism of generator and discriminator adversarial learning and is trained to generate and input images that are very similar. The conditional generative adversarial network (CGAN) came into being in order to ensure the safety of staff during image generation, and a large amount of research [34,35,36] based on different conditions was proposed. There have also been many studies on image generation that are based on different conditions, such as text-based [37,38], label-based [39], and image-based [40,41,42] conditions.

However, the generated image output by the above research work is generally distorted and the texture is not clear. Most models only repeat the content in the training set, and it is difficult to mine the semantic space. These models were trained on the DamperGenSet dataset, as the complex background information in DamperGenSet results in a mediocre quality of the generated images.

### 2.3. Research Summary

From the above research work, we can see that there is still room for improvement in vibration damper detection and image generation algorithms of overhead transmission lines. We summarize the following characteristics of these studies:

- Detection using traditional image processing algorithms is limited by the quality of the input image and the rationality of operator selection. If the background information in the image is too complex, the vibration damper will not be obvious enough, and importantly, the plated feature information will be weakened by the background. This makes it difficult for the feature operator to fully output the completed vibration damper information. The advantage of the traditional method is that the calculation speed is fast and the resource occupancy rate is low. In simple scenarios with high real-time requirements, it is still the most effective detection method.
- CNN-based methods are currently the most accurate solutions in the field of vibration damper detection. We only require a sufficient amount of training data to obtain an excellent end-to-end detection model. However, there is not yet a fully public vibration damper dataset. Nevertheless, such methods require higher computing power for operating equipment. We want to provide a solution that runs in real time on edge devices such as drones.
- The image generation algorithm led by GAN provides us with new solutions. CGAN uses the idea of adversarial learning to output high-quality images with a simple network structure. At the same time, auxiliary conditions can help us define the semantic information in the image. However, the current research work still has the problem of the image not being delicate enough, and there is no research work on the CGAN-based class of vibration dampers for transmission lines.

Therefore, combining the features summarized in the above research, we hope to obtain an end-to-end deep neural network model, one that can run in real time on edge devices such as drones. However, due to the difficulty of reproducing the actual scene of a transmission line in the data, the training data that the model relies on cannot be too high. Therefore, in this follow-up research work, we propose a model for generating vibration damper detection results on the basis of conditional generative adversarial networks.

## 3. Basic Knowledge of GAN

The generative adversarial networks (GANs) [12] are based on max–min game theory and consist of two sub-networks: a generator and a discriminator. The goal of the generator is to obtain a generated image that is very close to the original image with a d-dimensional noise. The discriminator scores images to decide whether the input is from a real image or a generated image. In a way, the discriminator exists for the generator to output high-quality images. On the other hand, the fidelity of the images output by the generator is constantly improving, and it is also constantly challenging the discriminative ability. The objective function of the entire GAN is as follows:

(1) $$\underset{G}{\min}\underset{D}{\max}V_{GAN}(G,D)=E_{x\sim p_{r}(x)}[\log D(x)]+E_{z\sim p_{g}(z)}[\log(1-D(G(z)))]$$

where x represents the real image obtained from the dataset and b represents a d-dimensional noise obtained using a normal distribution $p_{g}$.

On the basis of GAN, CGAN [13] introduces additional auxiliary variables to control the generation results of the model output. In CGAN, the generator uses auxiliary conditions to output generated images, and the discriminator uses auxiliary conditions as the basis to determine whether the input image is true or false. The objective function of CGAN is as follows:

(2) $$\underset{G}{\min}\underset{D}{\max}V_{CGAN}(G,D)=E_{s,x\sim p_{r}(s,x)}[\log D(s,x)]+E_{s,x^{\prime }\sim p_{r}(s,x^{\prime })}[\log(1-D(s,x^{\prime }))]$$

where s represents the auxiliary condition and $x^{\prime }=G(s,z)$ represents the output generated image.

In addition to the generator and discriminator losses, previous work [40,43] has shown that minimizing the distance between real and fake images can help the generator output higher-quality images.

Compared with the L2 distance, the L1 distance can more directly describe the difference between a real image and a fake image, and can also help generate images to reduce distortion and blurring problems.

Therefore, we also introduced the L1 distance in the model. The formula for calculating the L1 distance is as follows:

(3) $$\underset{G}{\min}V_{L1}(G)=E_{x,x^{\prime }\sim p_{r}(x,x^{\prime })}[{\left\|x-x^{\prime }\right\|}_{1}].$$

Therefore, the objective function of our model DamperGAN is the sum of (2) and (3).

## 4. DamperGAN

### 4.1. Overall Framework

**Figure 2:**
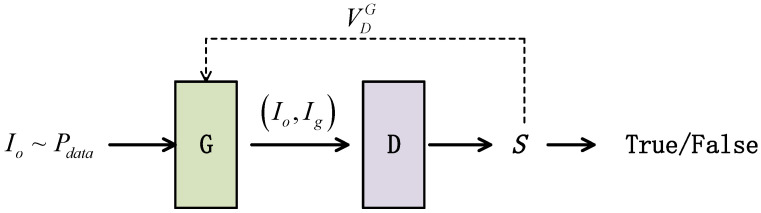
The framework of DamperGAN.

DamperGAN consists of a generator $G(I_{o};\theta_{g})$ and a discriminator $D((I_{o},I_{g});\theta_{d})$. We hope to use the generator G in DamperGAN to obtain a vibration damper detection image $I_{g}$ on the basis of the original image $I_{o}$ obtained by the drone. The overall structure of DamperGAN is shown in Figure 2, where $I_{o}$ represents the real dataset distribution, G represents the generator, D represents the discriminator, $I_{o}$ represents the original image, $I_{g}$ represents the generated image output by the generator, S represents the real image output by the discriminator degree score, and $V_{D}^{G}$ represents the calculated penalty value fed back to the generator.

The training of DamperGAN is divided into the learning of the generator and the learning of the discriminator, and the training of the two is a process of confrontation. The training goal of the generator is to output a high-quality fake image that is good enough to match the real image, while the goal of the discriminator is to discriminate between real and fake input images.

### 4.2. Multi-Granularity Generator

**Figure 3:**
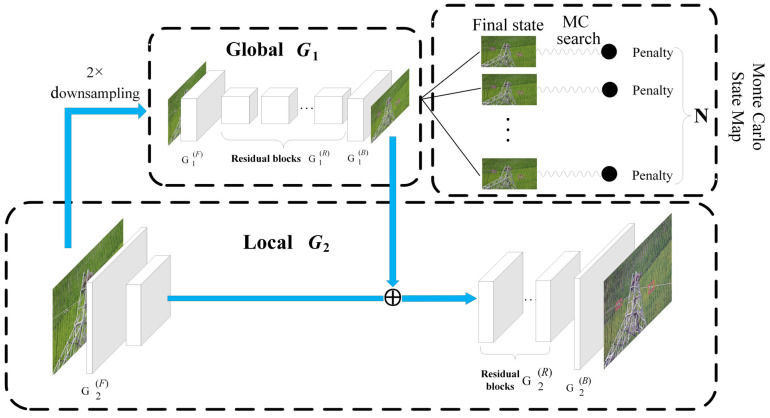
The framework of the generator containing three parts: the global module, the Monte Carlo search, and the local module. First, we used the global module to obtain the LR image; then, we used the Monte Carlo for detail mining; and finally, we used the local module to obtain the HR image.

The traditional generator in DamperGAN is decomposed into two sub-generators: $G_{1}$ and $G_{2}$, where $G_{1}$ is defined as a global generator and $G_{2}$ is defined as a local generator. The overall structure of the generator is shown in Figure 3. The local generator can effectively improve the resolution of the generated image. For example, the image with a resolution of 512 × 512 is input into the global generator, and the output resolution of the local generator is 1024 × 1024.

The global generator consists of three parts: front-end convolution layer $G_{1}^{\left(F\right)}$, residual block $G_{1}^{\left(R\right)}$, and transposed convolution back-end $G_{1}^{\left(B\right)}$. The original real images with a resolution of 512 × 512 are sequentially passed through these three parts, and the final output image with a resolution of 512 × 512 contains the dampers.

The local generator also contains three parts: front-end convolutional layer $G_{2}^{\left(F\right)}$, residual block $G_{2}^{\left(R\right)}$, and transposed convolution back-end $G_{2}^{\left(B\right)}$. The input of the local generator is the original image with a resolution of 1024 × 1024. Unlike the global generator, the input of the residual block of the local generator is the sum of the feature maps of the $G_{1}$ and the $G_{2}^{\left(F\right)}$, and thus, the extracted feature information from the global generator $G_{1}$ is passed to the local generator $G_{2}$.

In the training process, the original image of 1024 × 1024 was initially a down-sampling operation to obtain an image with a resolution of 512 × 512 to pre-train the residual network block of $G_{1}$ and then to train the local generator $G_{2}$, mainly because the resolution of two input images is different.

Additionally, in order to improve the semantic guidance for the generated images, we proposed an attention mechanism and a penalty mechanism based on Monte Carlo search.

#### 4.2.1. Penalty Mechanism

To further improve the details of the generated images and the positional accuracy of the boxes, we proposed a penalty mechanism based on a Monte Carlo search (MCS). Monte Carlo searches can mine latent spatial information from the data, and we can obtain the semantic details with clear texture on the basis of the results of the Monte Carlo search and the score of the discriminator.

We performed a Monte Carlo search on the basis of the results generated by the global generator $G_{1}$, and the search process can be expressed by the following equations:

(4) $$\left\{I_{g}^{1},\ldots ,I_{g}^{N}\right\}=MC^{G_{\beta }}\left(I_{g}^{\prime };N\right)$$

(5) $$I_{g}^{i}=G_{\beta }(I_{g}^{\prime },z)$$

where $I_{g}^{i}(i=1,\ldots ,N)$ represents the *N* images obtained on the basis of the results generated by the global generator $G_{1}$, and $MC^{G_{\beta }}$ represents the state we simulated using MCS. $G_{\beta }$ represents the generation model based on MCS virtualization technology that shares parameters with the local generator $G_{2}$. z∼N(0,1) represents the noise variable introduced during input that guarantees diversity in the search results.

In order to reward the search process with better results, we fed the *N* Monte Carlo search results and the generated images of the global generator $G_{1}$ output to the discriminator D, and we were able to obtain a penalty value $V_{D}^{G}$ according to the score output by the discriminator. The whole calculation process is shown as Equation (6).

(6) $$V_{D}^{G}=\frac{1}{N}\sum_{i=1}^{N}\left(\left(1-D(I_{g}^{i};\theta_{d}\right)\right)+{\left\|I_{g}-I_{t}\right\|}_{1}$$

where $D(I_{g}^{i};\theta_{d})$ represents the score of the image output from D, and the higher the score, the more likely it is to be a true image.

#### 4.2.2. Attention Mechanism

After obtaining multiple intermediate results through a Monte Carlo search, we hoped to use these results as the basis for the next high-resolution image generation task. We therefore introduced an attention mechanism to feed the positive effects of the Monte Carlo search into the local generator. We constructed a feature set with *N* intermediate results obtained by sampling and used the convolution operation to obtain different weight matrices for different results. Finally, the result obtained was fed into a local generator to obtain a higher-resolution target image.

We performed a convolution operation on the basis of *N* intermediate images to obtain the attention weight matrix. The results were calculated as shown in Equation (7).

(7) $$I_{A}^{i}=\mathrm{Softmax}(I_{g}^{i}W_{A}^{i}+b_{A}^{i}),\mathrm{for}\;i=1,\ldots ,N$$

where $I_{g}^{i}$ represents the input image, ${\left\{W_{A}^{i},b_{A}^{i}\right\}}_{i=1}^{N}$ represents the parameter of the convolution operation, and Softmax(·) represents the SoftMax function used for normalization. We multiplied the resulting attention weight matrix with the corresponding input image to obtain the final output:

(8) $$I_{g}^{\prime \prime }=(I_{A}^{1}⊗I_{g}^{1})⊕\ldots ⊕(I_{A}^{N}⊗I_{g}^{N})$$

where $I_{g}^{\prime \prime }$ represents the final output result of the attention mechanism; $I_{g}^{i}$ represents the input images; and the symbols ⊗ and ⊕ represent the multiplication and addition elements of the matrix, respectively.

#### 4.2.3. Objective Function

The loss function of the entire generator network is written as Equation (9).

(9) $$J_{G}(\theta_{g})=E_{I_{o}\sim P_{r}(I_{o}),I_{t}\sim P_{t}(I_{t})}\left[\log\left(1-D\left(I_{o},G\left(I_{o},I_{t}\right)\right)\right)\right]+V_{G}^{D}$$

where $D\left(I_{o},G\left(I_{o},I_{t}\right)\right)$ represents the score of the discriminator D for the image output by the generator G, which reflects the discriminator’s ability to discriminate against false images; $\theta_{g}$ represents the parameters constituting the generator; and $V_{G}^{D}$ represents the penalty value for the generated result output by Equation (6).

### 4.3. Multi-Level Discriminator

The structure of the discriminator is crucial for generating high-resolution images. The discriminator requires a deeper network or a larger-scale convolution kernel to extract feature information in order to distinguish between high-resolution real samples and generated samples, but this inevitably leads to a surge in network capacity and even overfitting. In addition, an overly complex discriminator will consume a large amount of memory resources, which are very scarce when generating high-resolution images.

#### 4.3.1. Multitasking Mechanism

**Figure 4:**
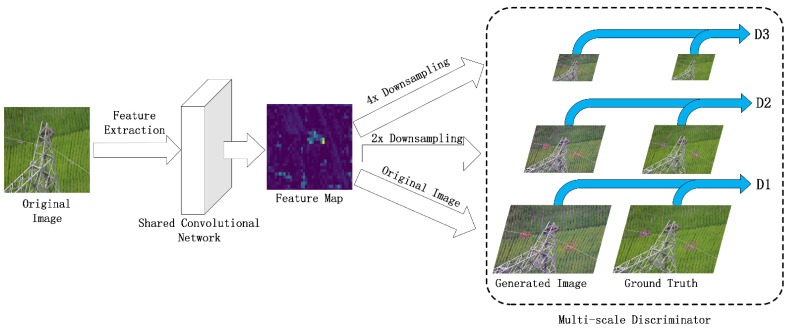
The discriminator consists of three parts with the same structure: *D*_1_, *D*_2_, and *D*_3_. First, we used the shared convolutional layer to obtain the feature map of input; then, the feature map was down-sampled for 2 times and 4 times and output to *D*_1_, *D*_2_, and *D*_3_; finally, we obtained the discriminator scores.

In order to improve the model’s ability to output high-resolution images, we proposed a multi-scale discriminator structure based on a multi-task learning mechanism. As shown in Figure 4, the network uses three discriminators—$D_{1}$, $D_{2}$, and $D_{3}$—with the same structure to deal with input images of different resolutions.

We utilized a multi-task learning strategy to train the discriminator on the basis of a parameter sharing mechanism [44]. First, we utilized the shared convolutional layers to obtain the feature maps of the samples. The feature map was then down-sampled with factors of 2 and 4 to obtain feature maps of the other two scales. Finally, three discriminators were used to process feature maps of three different scales.

Although the three discriminators use the same network structure, different inputs can bring about different discrimination capabilities to the entire discriminator network. The discriminator for small input can process more high-level semantic information, while the discriminator for large input can process more low-level feature information. Therefore, the structure of the multi-layer discriminator is very beneficial for improving the discrimination level of the entire discriminator network. When we deal with different identification requirements, we only need to increase or decrease the number of discriminators on the basis of the original model and do not need to retrain completely from scratch.

#### 4.3.2. Objective Function

The formula for the entire discriminator network is as shown in Equation (10).

(10) $$J_{D}(\theta_{d})=-\sum_{k=1,2,3}E_{I_{o}\sim P_{r}(I_{o}),I_{g}\sim P_{g}(I_{g})}\log D_{k}\left(\left(I_{o},I_{g}\right);\theta_{d}\right)$$

where $P_{r}$ represents the real dataset, $P_{g}$ represents the sample set of generated images, $I_{o}$ represents the original image, $I_{g}$ represents the generated image, $\theta_{d}$ represents the parameters of the discriminator network, and $D_{k}$ represents one of the discriminators.

On the basis of the entire multi-granularity generator and multi-scale discriminator proposed in this paper, we conducted adversarial learning of the two. The entire learning process is shown in Algorithm 1.
**Algorithm 1.** The training process of DamperGAN.Input: Real damper image dataset $I_{o}=\left\{I_{o_{1}},\ldots ,I_{o_{N}}\right\}$; Generator G; Discriminator    ${\left\{D_{i}\right\}}_{i=1}^{i=k}$; g-steps, the training step of the generator; d-steps, the training step of    the discriminators.Output: G, generator after training.1: Initialize generator G and discriminator ${\left\{D_{i}\right\}}_{i=1}^{i=k}$ with random weights;2: repeat3:  for g-steps, perform4:     G generate fake images;5:     Calculate the penalty value $V_{D}^{G}$ via Equation (6);6:     Minimize Equation (9) to update the parameters of the generator G;7:  end for8:  for d-steps, perform9:      Use G to generate fake images $I_{g}=\left\{I_{g_{1}},\ldots ,I_{g_{N}}\right\}$;10:    Use real images $I_{o}=\left\{I_{o_{1}},\ldots ,I_{o_{N}}\right\}$ and fake images $I_{g}=\left\{I_{g_{1}},\ldots ,I_{g_{N}}\right\}$ to update the discriminator parameters by minimizing Equation (10);11:  end for12: until DamperGAN completes convergence13: return

### 4.4. Network Structure

As we generated images on the basis of annotated images, there were a large number of low-level features between them. To improve the restoration of these features, we used a simple U-net [45] as the main infrastructure of the generator and discriminator.

The structure of the entire generator network is shown in Table 1. CONV stands for convolution operation, N-m indicates that the number of convolution kernels in each convolution layer is m; K-mxm indicates that the size of the kernel is mxm; S-m indicates that the stride of the kernel is m; P-m indicates that the size of the boundary expansion of the input image during the convolution operation is m, and IN, ReLU indicates that the current loss function is InstanceNorm-ReLU [46].

The structure of the entire discriminator network is shown in Table 2. Unlike the generator, InstanceNorm was not used for normalization in the first layer of the network, Convolution-InstanceNorm-LeakyReLU [47] was used as the loss function, and the slope of Leaky ReLU was 0.2. The convolutional layer used in the last layer produced a one-dimensional output, and the discriminator of the three-layer network architecture was the same.

## 5. Experiments and Analysis

### 5.1. Experiment Description

#### 5.1.1. Dataset

A dataset of vibration dampers for overhead transmission lines is required for the proposed theoretical validation and experimental analysis. At present, although there is a lot of research work on vibration dampers, there is no completely public vibration damper detection dataset. Moreover, most of the vibration damper data in the article were obtained by geometric transformation methods such as flipping, cutting, and scaling. An insufficient number of vibration dampers would make it difficult to verify the correctness of the proposed theory. Therefore, we made a dataset for vibration damper detection based on the real UAV cruise video of overhead transmission lines and named it DamperGenSet. In the process of making the DamperGenSet dataset, we used CAD2018 as a data labeling tool to label the positions of all existing line vibration dampers in the original image. The callout box used magenta and was as close as possible to the smallest bounding rectangle of the target area.

DamperGenSet contained a total of 3000 images, each of which contained vibration dampers, and the types of vibration dampers were not unique, such as hippocampus an-tislip vibration dampers and hook wire vibration dampers. We randomly divided all 3000 images into a training set and a test set. The training set contained 2500 images and the test set contained 500 images. The ratio of training set to test set was 5:1. In addition, as the dataset was obtained by shooting with UAVs, the presentation angle of the vibration damper in the image was not unique, which also put forward higher requirements for the robustness of the model.

#### 5.1.2. Experiment Configuration

In terms of hyperparameter settings in the experiment, we trained DamperGAN for a total of 200 epochs. The learning rate of the first 100 epochs remained unchanged, and the learning rate of the last 100 epochs gradually decreased to 0. In terms of experimental software settings, all our programs were written in Python language and integrated based on the PyTorch 1.4 platform. In the system environment of the experimental platform, we used Ubuntu18.04 as the operating system. In terms of the hardware environment of the experimental platform, we used an NVIDIA RTX 2080 GPU as the main equipment for training calculation, matched with an AMD R5-3600X CPU and 32 GB RAM.

### 5.2. The Baselines

In the following experiments, we use state-of-the-art methods of image generation as comparison methods.

Pix2Pix [40]: pix2pix is one of the most representative techniques in generative algorithms. The method uses an adversarial mechanism to learn the mapping between input and output, and has achieved excellent results in tasks such as image translation.

CRN [48]: This method is different from the adversarial training method in GAN, which just uses traditional convolutional layers to construct the entire network. It is worth noting that this method adds diversity loss between input and output to the loss function.

X-Fork [49]: X-fork is an image generation model that generates target images based on semantic segmentation maps and original images. The key to its effect is the quality of the semantic segmentation map.

X-Seq [49]: This method uses the idea of image generation to complete the task of semantic segmentation. The first generator outputs the image of the target viewpoint, and the second generator outputs the semantic segmentation map based on it.

SelectionGAN [50]: This method uses an attention mechanism to select the intermediate generated results of the model to improve the quality of the final generated pictures.

### 5.3. Qualitative Evaluation

**Figure 5:**
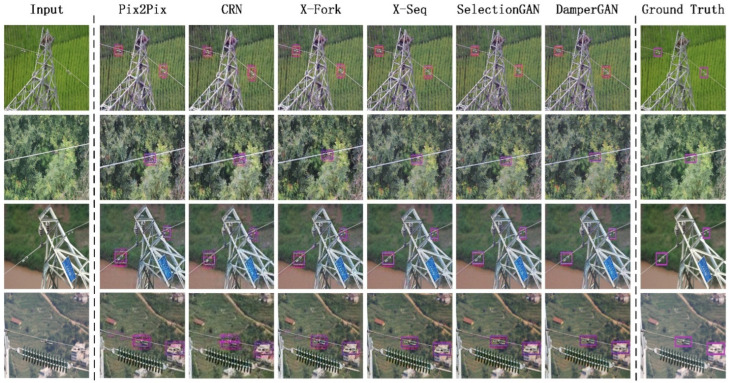
Test examples of each model on the DamperGenSet dataset.

To visually compare the difference between the detection effect of DamperGAN and other baselines, we conduct qualitative analysis and comparison experiments based on the DamperGenSet dataset. As can be seen from Figure 5, under the same test image, the detection effect of CRN is not stable enough. We believe that relying on convolutional layers for feature transfer alone is not enough to generate sufficiently realistic images. The performance of SelectionGAN is excellent. As a state-of-the-art framework based on CGAN, the attention mechanism provides a lot of reference information for the final result. The performance of pix2pix also has room for improvement. We argue that simple adversarial learning mechanisms still require other tricks to empower them. The entries for x-fork and x-seq are generally better. They all benefit from a multi-stage generation strategy, with different focuses in different generation stages, and task classification can make the whole network perform better. The image texture generated by the DamperGAN proposed in this paper is more refined, the position of the frame is more accurate, and the number of ghosts is lower. The overall performance of DamperGAN is better than other baselines.

### 5.4. Quantitative Evaluation

#### 5.4.1. Inception Score (IS) and Fréchet Inception Distance (FID)

The Inception Score (IS) is a common standard used to evaluate the quality of the output of the generative model, and the higher the value, the higher the clarity of the image. Its calculation formula is shown in Equation (11).

(11) $$IS(G)=\exp\left(E_{x\sim p_{g}}D_{KL}\left(p\left(y|x\right)∥p\left(y\right)\right)\right)$$

where x represents the generated image output by G, y represents the category of the generated image, $D_{KL}$ represents relative entropy, and $P_{g}$ represents the sample space of the generated image.

As the object we detected, the anti-vibration hammer, does not belong to the ImageNet dataset [51], we want to improve the test speed. Therefore, we use AlexNet instead of the Inception framework to score DamperGAN.

The Fréchet Inception Distance (FID) is a metric used to measure the gap between the generated image and the real image. The larger the value of FID, the less realistic the image is. Its calculation formula is shown in Equation (12).

(12) $$FID(I_{t},I_{g})={\left\|\mu_{I_{t}}-\mu_{I_{g}}\right\|}^{2}+T_{r}\left(\sum_{I_{t}}+\sum_{I_{g}}-2{\left(\sum {}_{I_{t}}\sum {}_{I_{g}}\right)}^{\frac{1}{2}}\right)$$

where $\mu_{I_{t}}$ represents the mean value of the feature map extracted from the original target image, $\mu_{I_{g}}$ represents the mean value of the feature map extracted from the generated image, $T_{r}$ represents the sum of the elements on the diagonal of the matrix, $\Sigma_{I_{t}}$ represents the covariance matrix of the feature map of the original image, and $\Sigma_{I_{g}}$ represents the covariance matrix of the image’s feature maps. We also use AlexNet instead of inception as the tool for extracting feature maps. We compute 4096 × 4096 covariance matrices $\Sigma_{I_{t}}$ and $\Sigma_{I_{g}}$ using the 1 × 4096 vector output from the last pooling layer.

As shown in Table 3, DamperGAN outperforms other baselines in IS and FID, and has the most improvement over classic pix2pix and CRN. This shows that the improvements in our proposed model provide additional reference information for image generation, and the obtained images are closest to the original in quality and similarity.

#### 5.4.2. Structural Similarity (SSIM), Peak Signal-to-Noise Ratio (PSNR), and Sharpness Difference (SD)

After evaluating the generative ability of the model using IS and FID, based on the research work in [52], we adopt three pixel-level metrics: SSIM, PSNR, and SD, to further evaluate the generated images.

Structural Similarity (SSIM) is an index that evaluates the similarity between images based on brightness and contrast. The value of SSIM ranges from −1 to 1, with larger values representing better performance. The formula for SSIM is shown in Equation (13).

(13) $$SSIM(I_{g},I_{t})=\frac{\left(2\mu_{I_{g}}\mu_{I_{t}}+c_{1})(2\sigma_{I_{g}I_{t}}+c_{2}\right)}{\left(\mu_{{}_{I_{g}}}^{2}+\mu_{I_{t}^{2}}^{2}+c_{1})(\sigma_{{}_{I_{g}}}^{2}+\sigma_{I_{t}^{2}}^{2}+c_{2}\right)}$$

where $I_{g}$ represents the output image, $I_{t}$ represents the standard image, $\mu_{I_{g}}$ represents the average value of the output image, $\mu_{I_{t}}$ represents the average value of the labeled image, $\sigma_{I_{g}}$ represents the standard deviation of the generated image, $\sigma_{I_{t}}$ represents the standard deviation of the labeled image, and $c_{1},c_{2}$ are the adjustment factors.

The peak signal-to-noise ratio (PSNR) uses the peak signal of the noise ratio between the real image and the generated image for similarity comparison. Higher numbers represent better similarity. The calculation formula of PSNR is shown in Equations (14) and (15), and the specific meaning of the parameters is the same as above.

(14) $$PSNR(I_{g},I_{t})=10\log_{10}\left(\frac{\max_{I_{g}}^{2}}{mse}\right)$$

(15) $$mse(I_{g},I_{t})=\frac{1}{n}\sum_{i=1}^{n}{\left(I_{t}\left[i\right]-I_{g}\left[i\right]\right)}^{2},\max_{I_{g}}=255$$

The sharpness difference (SD) is a metric used to describe the degree of sharpness loss during image generation, and we refer to the work in [52] to describe this criterion by computing the gradient change between the generated image and the original image. The calculation formula of SD is shown in Equations (16) and (17), with the specific meaning of the parameters being the same as above.

(16) $$\mathrm{SharpDiff}.(I_{g},I_{t})=10\log_{10}\left(\frac{\max_{I_{g}}^{2}}{grads}\right)$$

(17) $$grads=\frac{1}{N}\sum_{i}\sum_{j}\left(\left|\left(\nabla_{i}I_{v}+\nabla_{j}I_{v}\right)-\left(\nabla_{i}I_{g}+\nabla_{j}I_{g}\right)\right|\right)$$

where $\nabla_{i}I=\left|I_{i,j}-I_{i-1,j}\right|,\nabla_{j}I=\left|I_{i,j}-I_{i,j-1}\right|$.

As can be seen from Table 4, under the same test picture, thanks to the attention mechanism, the performance of SelectionGAN is still stable, and its performance under various standards is in the forefront; however, its good score comes at the cost of great computation time. The X-Fork and X-Seq perform similarly, both of them having a certain degree of lead in terms of indicators. In addition, the calculation speed is faster than SelectionGAN. The Pix2pix and CRN have the lowest score. However, the advantage of them is that the calculation speed is much faster than other baselines, which is a significant advantage for scenarios with extremely high real-time requirements. DamperGAN outperforms other baselines in all three evaluation indicators, and the performance on FPS is also similar to SelectionGAN. We believe that two-stage generation, Monte Carlo search, etc. allow it to have better performance and use less additional computational cost.

### 5.5. Sensitivity Analysis

In this section, we perform multiple sets of sensitivity analysis on each component of DamperGAN, which includes the choice of backbone, edge extraction, attention mechanism, number of training iterations, and minimum amount of training data.

#### 5.5.1. Two-Stage Generation

We conduct a sensitivity analysis on the generator used by DamperGAN while retaining other improvements. As shown in Table 5, the performance of using a two-stage generator is most balanced, the performance of a single-stage generator is average, and the time-consumption of a three-stage generator is too high.

#### 5.5.2. Monte Carlo Search

The Monte Carlo search is the basis of the attention mechanism and the penalty mechanism. The introduction of Monte Carlo search allows us to further mine semantic information on the basis of low-resolution generated images and improve the basis for high-resolution generation. Therefore, the number of Monte Carlo searches that are introduced is critical to the performance impact. We conduct a comparative analysis on the number of Monte Carlo searches introduced. As shown in Table 6, when the number of Monte Carlo searches introduced is 5, the model can achieve a balance between the quality of the output results and the calculation speed.

#### 5.5.3. Multi-Level Discriminator

In order to cope with the excellent performance of two-stage generation, we use the multi-task learning mechanism to propose a multi-level discriminator structure to improve the discriminator’s discriminative ability, which will further improve the effect of the generator’s output image. Therefore, the number of layers of the multi-layer discriminator affects the performance of the entire network. As shown in Table 7, when the number of comparators with the same structure is 3, a good result can be achieved between model performance and calculation speed.

#### 5.5.4. Number of Epochs

The number of epochs for experimental training will affect the performance of the model. As the number of training epochs is not enough, the model will be under-fitted, and the model has not yet fully learned to identify all the objects to be detected. Excessive training epochs will reduce the robustness of the model, the parameters are limited by the existing training data, and the realization of unfamiliar data in the test set will be reduced. Therefore, we conduct an evaluation test of the number of training times for the performance of the model, and the test results are shown in Table 8. When the training epoch is 200, the model performs the most balanced.

#### 5.5.5. Minimum Training Data Experiment

Changes in the amount of training data will also affect the final performance of the model. At the same time, by comparing the performance of the model under different amounts of data, we can determine the generation ability of the model. As shown in Table 9, we conduct experiments with the minimum amount of data. The model performance does not drop significantly until the test set drops to 1750. Moreover, DamperGAN has strong robustness and can still learn key feature information on small-scale datasets, which overcomes the shortcomings of the previous model’s poor generalization ability to a certain extent.

### 5.6. Computational Complexity

The network parameters and training time are recorded to evaluate the space and time complexity of the networks. As shown in Table 10, compared with SelectionGAN, DamperGAN has a similar performance, but its parameters and training time are reduced. Overall, our model outperforms other baselines, which translates to increased temporal and spatial occupancy. However, the consumption of these resources is worth it, because we obtain the highest test results, and the FPS can support the requirements of real-time operation.

## 6. Conclusions

This paper proposes a power line vibration damper detection image-generation model named DamperGAN based on CGAN, which can detect the position of the vibration damper in drone inspection aerial images. DamperGAN first generates low-resolution images based on coarse-grained modules, uses Monte Carlo search to mine latent information in low-resolution images, and then uses the attention mechanism to introduce positive information into fine-grained modules to output high-resolution images, using the penalty mechanism to evaluate the state of the mined intermediate information to improve the convergence effect of the model. We draw the following conclusions through qualitative and quantitative experiments on the self-built DamperGenSet dataset. The detection images generated by DamperGAN are closest to the ground truth in detail texture. Our model outperforms other baselines under multiple evaluation metrics. Sensitivity analysis experiments show that the two-stage generation, Monte Carlo search, and multi-level discriminator utilized in the model all have a positive impact on the final performance. All experimental results show that DamperGAN has the ability to detect vibration dampers in real time using a UAV, which provides a solid foundation for tasks such as corrosion and displacement detection of vibration dampers. In the future, we will continue to explore feasible optimization schemes for DamperGAN and incorporate more power device detection tasks.

## Figures and Tables

**Table 1:** The architecture of the generator.

Network	Layer Information	Input	Output
Down-Sample	CONV-(N64, K7 × 7, S1, P3), IN, ReLU	(512, 512, 3)	(512, 512, 64)
	CONV-(N128, K3 × 3, S2, P1), IN, ReLU	(512, 512, 64)	(256, 256, 128)
	CONV-(N256, K3 × 3, S2, P1), IN, ReLU	(256, 256, 128)	(128, 128, 256)
	CONV-(N512, K3 × 3, S2, P1), IN, ReLU	(128, 128, 256)	(64, 64, 512)
	CONV-(N1024, K3 × 3, S2, P1), IN, ReLU	(64, 64, 512)	(32, 32, 1024)
Residual Block	CONV-(N1024, K3 × 3, S1, P1), IN, ReLU	(32, 32, 1024)	(32, 32, 1024)
	CONV-(N1024, K3 × 3, S1, P1), IN, ReLU	(32, 32, 1024)	(32, 32, 1024)
	CONV-(N1024, K3 × 3, S1, P1), IN, ReLU	(32, 32, 1024)	(32, 32, 1024)
	CONV-(N1024, K3 × 3, S1, P1), IN, ReLU	(32, 32, 1024)	(32, 32, 1024)
	CONV-(N1024, K3 × 3, S1, P1), IN, ReLU	(32, 32, 1024)	(32, 32, 1024)
	CONV-(N1024, K3 × 3, S1, P1), IN, ReLU	(32, 32, 1024)	(32, 32, 1024)
	CONV-(N1024, K3 × 3, S1, P1), IN, ReLU	(32, 32, 1024)	(32, 32, 1024)
	CONV-(N1024, K3 × 3, S1, P1), IN, ReLU	(32, 32, 1024)	(32, 32, 1024)
	CONV-(N1024, K3 × 3, S1, P1), IN, ReLU	(32, 32, 1024)	(32, 32, 1024)
Up-Sample	CONV-(N512, K3 × 3, S0.5, P1), IN, ReLU	(32, 32, 1024)	(64, 64, 512)
	CONV-(N256, K3 × 3, S0.5, P1), IN, ReLU	(64, 64, 512)	(128, 128, 256)
	CONV-(N128, K3 × 3, S0.5, P1), IN, ReLU	(128, 128, 256)	(256, 256, 128)
	CONV-(N64, K3 × 3, S0.5, P1), IN, ReLU	(256, 256, 128)	(512, 512, 64)
	CONV-(N3, K7 × 7, S1, P3), IN, ReLU	(512, 512, 64)	(512, 512, 3)

**Table 2:** The architecture of the discriminators.

Network	Layer Information	Input	Output
Input Layer	CONV-(N64, K4 × 4, S2, P2), Leaky ReLU	(512, 512, 3)	(256, 256, 64)
	CONV-(N128, K4 × 4, S2, P2), IN, ReLU	(256, 256, 64)	(128, 128, 128)
	CONV-(N256, K4 × 4, S2, P2), IN, ReLU	(128, 128, 128)	(64, 64, 256)
	CONV-(N512, K4 × 4, S2, P2), IN, ReLU	(64, 64, 256)	(32, 32, 512)

**Table 3:** IS and FID of the different models.

Model	InsuGenSet	
	IS	FID
Pix2Pix	3.25	57.45
CRN	3.04	57.68
X-Fork	3.37	56.90
X-Seq	3.61	56.42
SelectionGAN	3.75	56.08
DamperGAN	3.83	55.31

**Table 4:** SSIM, PSNR, SD, and FPS of the different models.

Model	InsuGenSet			FPS
	SSIM	PSNR	SD	
Pix2Pix	0.29	15.91	17.41	160
CRN	0.27	15.53	17.12	187
X-Fork	0.38	16.37	18.21	85
X-Seq	0.45	17.34	18.58	72
SelectionGAN	0.63	26.83	20.61	66
DamperGAN	0.70	28.14	22.13	63

**Table 5:** Comparison of the effectiveness of the generator networks.

	IS	FID	FPS
Single generator	3.28	56.84	82
Two-stage generator	3.83	55.31	63
Three-stage generator	4.25	54.96	37

**Table 6:** Introducing the Monte Carlo search time comparison.

MCS	SSIM	PSNR	SD	FPS
Not introduced	0.57	26.28	19.30	75
*N* = 1	0.63	26.84	20.46	70
*N* = 3	0.68	27.60	21.37	67
*N* = 5	0.70	28.14	22.13	63
*N* = 7	0.72	28.47	22.54	58
*N* = 9	0.73	28.62	23.02	51

**Table 7:** Comparison of the effectiveness of the discriminant networks.

	SSIM	PSNR	SD	FPS
Single discriminator	0.60	26.48	19.84	71
Two-level discriminator	0.65	27.26	20.62	67
Three-level discriminator	0.70	28.14	22.13	63
Four-level discriminator	0.72	28.53	22.79	58

**Table 8:** The effect of different epoch numbers on the experimental results.

Number of Epochs	SSIM	PSNR	SD	FPS
50	0.32	16.72	17.84	65
100	0.58	18.56	19.05	64
150	0.64	24.47	21.31	63
200	0.70	28.14	22.13	63
250	0.68	27.92	21.86	61

**Table 9:** Minimum training data experimental results.

The Amount of Training Set	SSIM	PSNR	SD
2500 (100%)	0.70	28.14	22.13
2250 (90%)	0.68	27.82	21.94
2000 (80%)	0.65	25.86	20.25
1750 (70%)	0.62	25.15	19.93
1500 (60%)	0.56	23.83	17.42

**Table 10:** Network parameters (Param.) and training time of the different models.

Model	Param.	Training Time (h)
Pix2Pix	47 M	14.92
CRN	36 M	10.88
X-Fork	62 M	16.30
X-Seq	70 M	18.57
SelectionGAN	78 M	20.06
DamperGAN	82 M	22.68

## Data Availability

The data in this paper are undisclosed due to the confidentiality requirements of the data supplier.
